# The structure-function relationship of disulfide bonds in etanercept

**DOI:** 10.1038/s41598-017-04320-5

**Published:** 2017-06-21

**Authors:** William C. Lamanna, Robert Ernst Mayer, Alfred Rupprechter, Michael Fuchs, Fabian Higel, Cornelius Fritsch, Cornelia Vogelsang, Andreas Seidl, Hansjoerg Toll, Martin Schiestl, Johann Holzmann

**Affiliations:** 1 Sandoz Biopharmaceuticals, Sandoz GmbH, Biochemiestraße 10, 6250 Kundl, Austria; 2 Technical Development Biosimilars, Biologics Technical Development and Manufacturing, Novartis, Sandoz GmbH, Biochemiestraße 10, 6250 Kundl, Austria; 3 Technical Development Biosimilars, Biologics Technical Development and Manufacturing, Novartis, Hexal AG, Keltenring 1+3, 82041 Oberhaching, Germany; 4 Novartis Pharma AG, Klybeckstrasse 141, CH-4057 Basel, Switzerland

## Abstract

Etanercept is a TNFα receptor Fc fusion protein used for the treatment of rheumatic disease and psoriasis. Physicochemical and functional investigation of process fractions during development of the etanercept biosimilar GP2015 (Erelzi^®^) revealed a correlation between reduced potency and incorrect disulfide bridging between specific cysteines in the receptor domain. This novel structure-function relationship was found to be the molecular basis for reduced potency in recent Enbrel^®^ batches, which exhibit higher levels of incorrect disulfide bridging. Interestingly, incorrect disulfide bridging was found to be reversible under serum-like redox conditions, restoring potency to normal levels. This redox dependent reversibility suggests that these variants are likely not relevant for clinical efficacy once the drug enters the bloodstream. Nonetheless, incorrect disulfide bridging in etanercept represents a new quality attribute that is critical for biopharmaceutical functionality and should thus be carefully monitored and controlled to guarantee patient safety.

## Introduction

Tumor necrosis factor alpha (TNFα) is a potent cytokine involved in immune response regulation and elicitation of inflammatory response^1, 2^. The protein is produced by cells as a 26 kDa membrane bound precursor (mTNFα) that can be subsequently processed by cell surface metalloproteinase known as TNFα converting enzyme (TACE) or ADAM 17 to generate a soluble 17 kDa form (sTNFα). Both membrane bound and soluble TNFα can bind and activate TNF receptors 1 or 2 to elicit pro-inflammatory cytokine cascades, tissue destruction and cell death, which, if exacerbated, can result in a variety of inflammatory, rheumatic or dermatological diseases^3–6^. The development of biopharmaceutical drugs, such as etanercept, that can specifically and potently bind and neutralize TNFα have dramatically improved the ability to treat a number of pathological conditions including rheumatoid arthritis, ankylosing spondylitis and psoriasis^7–9^.

Etanercept is a ~125 kDa fusion protein, consisting of a TNF receptor 2 domain coupled to the Fc portion of human IgG_1_^10^ (data not shown). Each amino acid chain of the molecular dimer contains three N-glycans as well as up to fourteen sites that can be differentially modified by O-glycosylation^11^ (data not shown). Further, the etanercept molecule contains thirteen intra-chain and three inter-chain disulfide bonds, the majority of which are located in the TNF receptor region of the molecule^12, 13^. The size and complexity of biopharmaceuticals such as etanercept present an exceptional challenge for companies aiming to produce follow on biosimilar products, which must comprise highly similar structural and functional properties and exhibit no clinically meaningful differences to the original reference prodcut^14–16^. To achieve this regulatory standard, extensive physicochemical and functional characterization of both the biosimilar and the reference product is required to understand the product characteristics and to allow targeted control of variants that impact the structure and function of the molecule.

**Figure 1:**
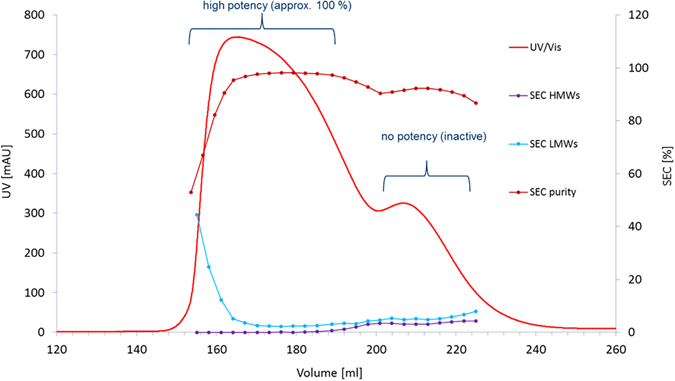
Potency loss in late eluting large scale HIC fractions. Large scale HIC separation of GP2015 (Erelzi^®^) resulted in the separation of a main peak from a late eluting shoulder peak. Assessment of potency in these two fractions revealed near 100% in the main peak while the late eluting fraction was found to be inactive. Although high and low molecular weight variants were slightly enriched in the late eluting fraction, this moderate decrease in purity could not explain the loss of potency.

In the course of process development for the etanercept biosimilar GP2015 (Erelzi^®^) at Sandoz a loss of potency, as measured by TNFα inhibition reporter gene assay, was observed in late eluting fractions during hydrophobic interaction chromatography (HIC) purification (see Fig. 1). This loss of potency could not be correlated with product related variants known to influence etanercept potency, such as low molecular weight degradation products (LMW variants) or high molecular weight aggregates (HMW variants), which were only moderately enriched in the late eluting fraction (see Fig. 1).

**Figure 2:**
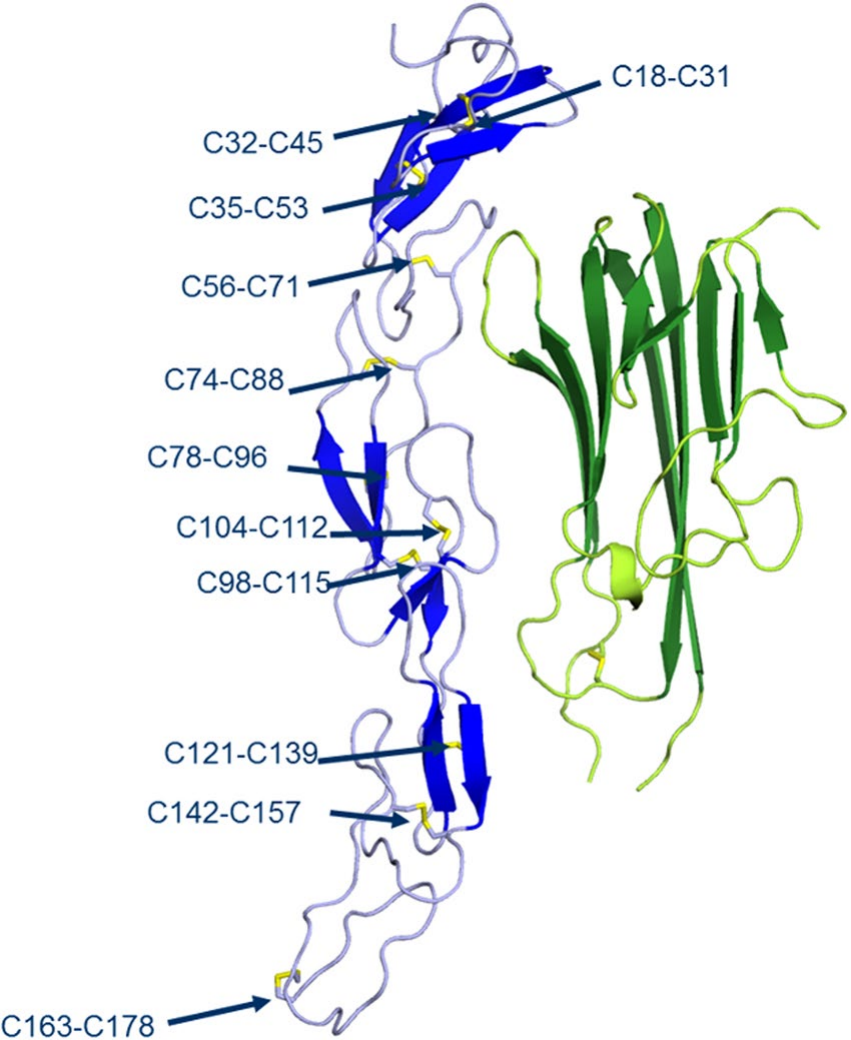
Disulfide bridge structures in etanercept. X-ray crystallography of the GP2015 TNF-receptor domain (blue) in conjugation with TNFα (green) was achieved and provides important structural information for etanercept. The eleven disulfide bridge structures in the receptor domain are in line with those previously reported in the literature for crystal structures of etanercept^12^. The LC-MS based triple digest peptide map (see methods for details) was able to identify each of the eleven disulfide bridge structures as well as four additional incorrect disulfide bonds between cysteines C_18_-C_74_, C_71_-C_88_, C_78_-C_88_ and C_71_-C_74_ in both GP2015 (Erelzi^®^) and the reference product (Enbrel^®^).

The TNF receptor domain of etanercept is stabilized by eleven disulfide bonds. While no direct evidence demonstrating an impact of incorrect disulfide bridging on potency has yet been reported, there has been speculation based on similar observations that late eluting HIC fractions exhibit low potency without observable aggregation, degradation or modification^13, 17–20^. To characterize disulfide bridging in GP2015 (Erelzi^®^) and Enbrel^®^ samples, an LC-MS based native peptide map approach using three distinct proteolytic enzymes was developed (‘triple digest’, see methods and Supplementary Table S1 for details). Using this “triple digest” assay, we identified each of the eleven intra-chain disulfide bonds in the receptor region of etanercept, which correspond with those identified by X-ray crystallography^12^ (see Fig. 2). In addition, four incorrect disulfide bridged variants, C_18_-C_74_, C_71_-C_88_, C_78_-C_88_ and C_71_-C_74_, were identified which have not yet been reported in the literature. These incorrect disulfide bonds all localize to the TNFα binding region of etanercept (see Supplementary Fig. S1 for details) and were ubiquitously present in all GP2015 (Erelzi^®^) and Enbrel^®^ batches.

To assess whether incorrect disulfide bridging directly impacts etanercept potency, we developed a non-reducing peptide map approach for quantitation of the incorrectly bridged C_78_-C_88_ peptide relative to an internal peptide standard^21, 22^ (see methods section for details). Compared to the other three incorrect disulfide bridges, quantitation of the peptide containing C_78_-C_88_ was found to provide the most accurate and repeatable results, likely due to the robust chromatographic resolution of the relevant peptide and its refraction to misscleavage or modification during sample preparation (see Supplementary Tables S2 and S3 for details). To allow accurate C_78_-C_88_ quantitation, the non reducing peptide map was developed under conditions which repress disulfide shuffling^23, 24^. This was achieved in part by using a mild detergent (RapiGest SF) in the presence of iodoacetamide during the denaturing step to quench nonspecific reactions with unpaired disulfide bonds. In addition, the enzymatic digestion was carried out under moderately acidic conditions which were found to stably suppress disulfide bridge shuffling to approximately 1% in GP2015 (Erelzi^®^) drug substance samples (see Table 1).

**Table 1:** The impact of pH on disulfide bridge shuffling.

	pH 8.0	pH 7.5	pH 6.5	pH 5.5	pH 5.0
GP2015 DS	11.3%	9.5%	6.7%	1.3%	1.2%
GP2015 CAP.E	15.5%	12.3%	8.1%	3.9%	4.3%

The table above shows the relative amount of peptide containing the incorrect disulfide bridge C_78_-C_88_ in GP2015 drugs substance (DS) samples and GP2015 capture eluate process intermediates (CAP.E) when the digestion step of the non reducing peptide map was carried out at different pH. Higher pH was found to results in high levels of C_78_-C_88_, while the level of internal peptide standard remained stable. These data suggest that increased pH promotes disulfide bridge shuffling and results in increased incorrect bridging. In contrast, performance of the non reducing peptide map at pH 6.0 or below resulted in stable low levels of C_78_-C_88_ peptide suggesting a repression of shuffling.

**Figure 3:**
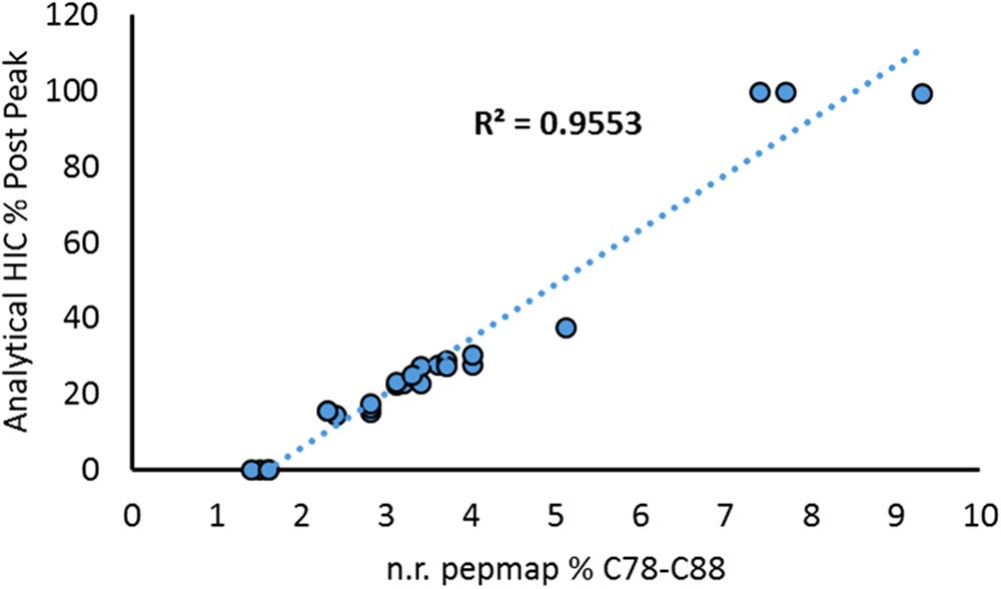
Approximation of overall incorrect disulfide bridging using analytical hydrophobic interaction chromatography. Samples with different potencies, such as GP2015 (Erelzi^®^) drug substance, process intermediates and late eluting HIC fractions were analyzed using the analytical HIC and the non reducing peptide map methods. The abundance of incorrect disulfide bridging assessed by these methods was found to correlate, allowing approximation of how the relative amount of C_78_-C_88_ assessed by the non reducing peptide map corresponds with overall incorrect disulfide bridging in etanercept, as reflected by the HIC post peak.

To quantitatively assess how the relative abundance of incorrectly bridged C_78_-C_88_ correlates with the overall amount of incorrect disulfide bridging in the etanercept molecule, an analytical HIC method was additionally developed which chromatographically separates incorrectly bridged structures based on changes in secondary and tertiary structure. Etanercept variants containing incorrect disulfide bridge structures were found to elute as a post peak in the analytical HIC together with high molecular weight aggregates. The correlation of non reducing peptide map and HIC results can be used to approximate how the relative percent C_78_-C_88_ peptide values translates into the overall amount of incorrect disulfide bridging present in a sample (see Fig. 3). Based on these results, samples with 1% C_78_-C_88_ are essentially free of incorrectly bridged structures, and can thus be considered artifact level incorrect bridging generated by residual shuffling of disulfide bridges during sample preparation. Samples containing 2% C_78_-C_88_ contain approximately 10% incorrectly bridged structures as measured by HIC. As described below, the percent incorrectly bridged structures estimated using the analytical HIC values correspond to the relative loss of potency, suggesting a direct correlation between incorrect disulfide bridging and potency loss.

**Figure 4:**
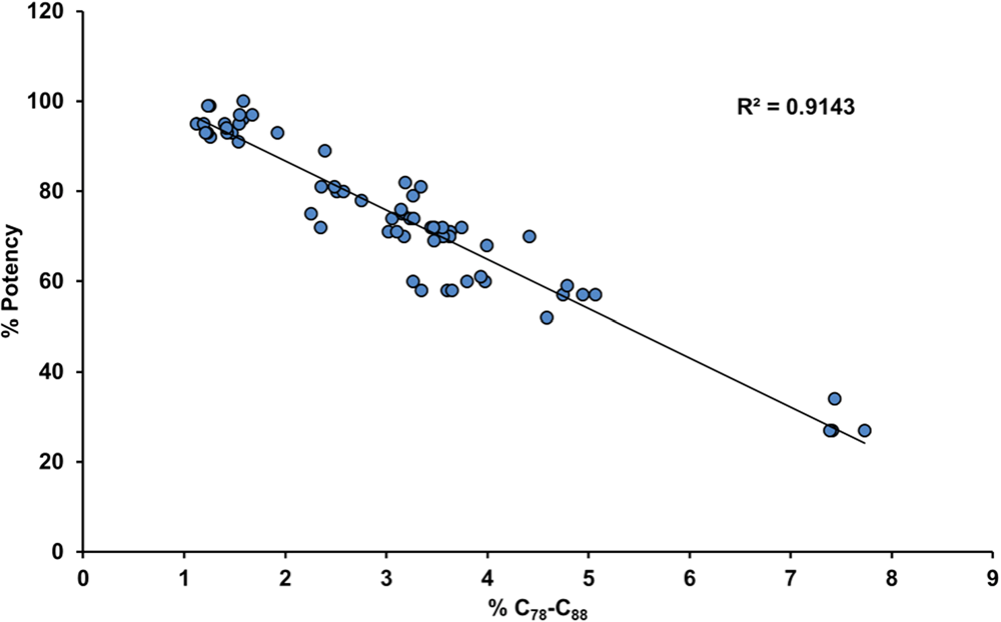
Correlation of incorrect disulfide bridge C_78_-C_88_ and potency. The relative amount of C_78_-C_88_ was quantified by non reducing peptide map analysis in different samples including reference product (Enbrel^®^), GP2015 (Erelzi^®^), process intermediates and late eluting HIC fractions. Evaluation of incorrect disulfide bridging relative to potency in these samples revealed a strong inverse correlation between potency and the relative abundance of C_78_-C_88_.

To assess whether a real causation between incorrect disulfide bridging and potency exists, the C_78_-C_88_ peptide was quantified in an array of GP2015 process intermediates, late eluting HIC fractions and reference product samples exhibiting a wide range of potencies. As shown in Fig. 4, a strong inverse correlation was observed between potency and the abundance of the peptide containing C_78_-C_88_, indicating a 10% decrease in potency for every 1% increase in the relative amount of incorrectly bridged variant. The functional relationship between C_78_-C_88_ abundance and etanercept potency is underlined by the high correlation value. The high corollary impact of this incorrectly bridged variant on etanercept function has important implications for companies seeking to market biosimilar versions of this molecule, as matching abundance of incorrectly bridged C_78_-C_88_ peptide in biosimilar and the reference products must be considered essential for guaranteeing comparable potency.

It has been noted in the literature that redox conditions, mimicking those found in human serum, can result in disulfide bridge reshuffling into energetically stable and correct structures^23, 25–27^. To assess whether serum-like redox conditions can result in the reshuffling of incorrectly bridged variants in etanercept, selected process intermediate GP2015 and reference product samples containing higher amounts of C_78_-C_88_, were incubated with physiologically relevant levels of cysteine for 48 hours at low temperature (see methods for details). As shown in Table 2, incubation under physiological redox conditions mediated an almost complete elimination of incorrectly bridged C_78_-C_88_ variant, resulting in subsequent restoration of potency. Following redox treatment, the adjusted levels of incorrectly bridged variant and potency continued to correlate along the linear regression, indicating that the observed improvements in potency are mediated exclusively through the correction of disulfide bridging (data not shown). These results suggest that while the abundance of incorrectly bridged C_78_-C_88_ predicts the degree of potency *in vitro*, *in vivo* efficacy would likely not be impacted by this incorrect disulfide bridging due to reshuffling once the drug is exposed to physiological redox conditions in the blood stream.

**Table 2:** The impact of serum-like redox conditions on incorrect disulfide bridging and potency.

Sample (batch number)	% C_78_–C_88_ (untreated)	% C_78_–C_88_ (redox)	% Potency (untreated)	% Potency (redox)
GP2015 DP (30986553)	1.2	1.5	98	103
GP2015 DP (30986557)	1.8	1.3	97	101
GP2015 DP (31161503)	1.2	1.7	100	98
GP2015 DS (B280815)	1.0	1.2	99	103
Enbrel^®^ (J13793)	2.3	1.6	92	100
Enbrel^®^ (1040542)	2.6	1.7	89	107
Enbrel^®^ (1062728)	2.5	1.8	85	98
Enbrel^®^ (1034018)	2.8	1.8	81	96
Enbrel^®^ (1034842)	2.5	1.8	85	95
GP2015 process intermediate (CAP.E)	3.4	1.6	76	98
GP2015 late eluting HIC fraction	5.5	2.0	58	93
		**1.6**	Mean	**99**
		**14**	RSD	**4**

The degree of potency and the relative amount of C_78_-C_88_, as determined by non reducing peptide map analysis, was assessed in selected GP2015 (Erelzi^®^) and Enbrel^®^ reference product samples before and after exposure to serum-like redox conditions (see methods section for details). Following exposure, a concomitant elimination of incorrect disulfide bridging and restoration of potency was observed in all samples. Mean values of C_78_-C_88_ and potency following redox treatment are indicated along with the relative standard deviation (RSD).

**Figure 5:**
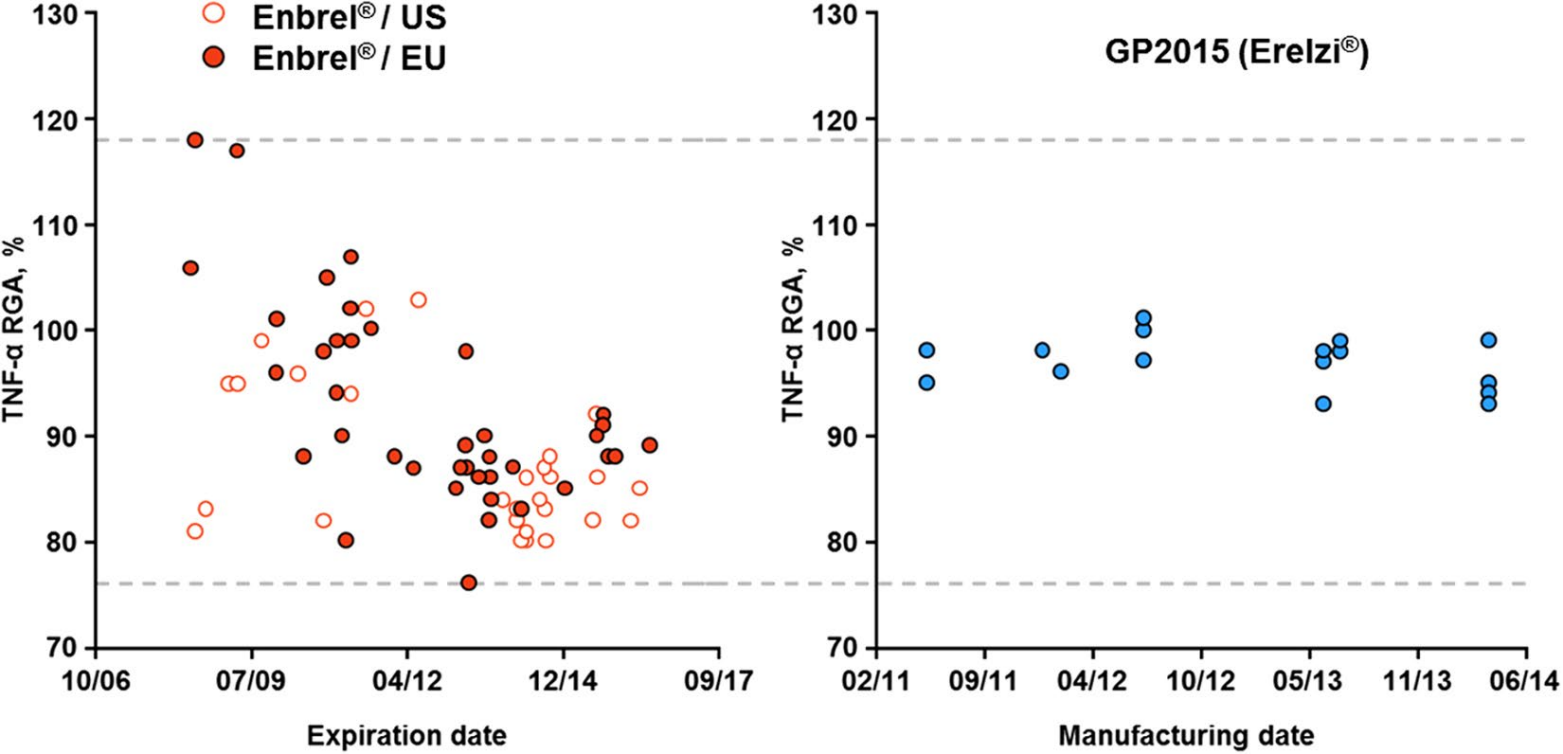
Assessment of potency variability over time. Assessment of potency in reference product Enbrel^®^ batches sourced from both the EU and US since 2008 reveals a broad distribution with a tightening of values toward the lower end over time, likely due to one or more process changes. Assessment of potency for GP2015 (Erelzi^®^) batches over time reveals low batch to batch variability well within the clinically verified range of the reference product. Manufacturing dates are given as month/year.

Assessment of reference product Enbrel^®^ batches over time revealed reduced potency levels in more recent batches, likely resulting from a process change (see Fig. 5). This tightening of potency values at the lower range of the overall batch to batch variability was found to correlate with a consistently higher relative amount of incorrectly bridged C_78_-C_88_ variant in selected batches. Manufacturing process changes for biopharmaceuticals are a common occurrence and can impact the type and abundance of quality attributes critical for protein structure and function^28–30^. Reference product process changes are particularly relevant for biosimilar companies whose aim is to develop a follow on product with the same safety and efficacy profile. Indeed, biosimilar development normally begins with the analysis of multiple batches of the reference product to define its batch to batch variability and to establish the reference product target ranges. These target ranges are the basis for selection of an appropriate production cell clone and for the development of a manufacturing process that delivers a highly similar product with matching structural and functional properties. Therefore, even in cases where the biosimilar candidate is indistinguishable from the initially tested reference product batches, post approval process changes carried out by the reference product company can result in measurable analytical differences in some quality attributes. Importantly, health authorities approve process changes only if they do not result in clinically meaningful differences. To achieve this, they apply the same principles for evaluation of comparability that govern an evaluation of biosimilarity, assuring through extensive analytical and, if necessary, confirmatory clinical testing, that pre- and post-change batches exhibit the same safety and efficacy^31^. Thus, regardless of whether a reference product manufacturer implements process changes, demonstrating that functionally critical structural attributes of a biosimilar remain within the clinically verified range of the reference product is central to assuring the same safety and efficacy profile. As shown in Fig. 5, the Sandoz biosimilar etanercept GP2015 (Erelzi^®^) was developed with consistently low levels of incorrectly bridged variant, resulting in stable potency levels with low batch to batch variability that lie well within the overall clinically verified range of the reference product batches.

Overall, this paper describes the characterization of a novel structure-function relationship between disulfide bonding and potency in etanercept. All batches of the recently FDA approved etanercept biosimilar GP2015 (Erelzi^®^) were found to contain identical incorrect disulfide bridge structures as Enbrel^®^, at levels within the clinically verified range of the reference product. Using a quantitative non reducing peptide map method, the relative amount of incorrectly bridged C_78_-C_88_ peptides could be inversely correlated with the degree of potency in GP2015 (Erelzi^®^) and Enbrel^®^ samples. This approach was used to explain a potency shift in reference product Enbrel^®^, which could be associated with increased levels of incorrect disulfide bridging at C_78_-C_88_. Interestingly, incorrect disulfide bridging in etanercept was found to be reversible under redox conditions similar to those found in serum, suggesting that while critical for potency *in vitro*, such variants are likely not relevant for efficacy *in vivo*. We believe the insights and methodologies described in this manuscript will be important for pharmaceutical companies seeking to manufacture biosimilar versions of etanercept, as these companies cannot rely on long term manufacturing experience but instead must leverage detailed knowledge of quality attributes critical for molecular function to specifically control related variants and ensure that product safety and consistency are maintained. The functional relationship between incorrect disulfide bridging and potency demonstrated in this manuscript suggests that companies seeking to produce biosimilar versions of etanercept should establish comprehensive characterization and control of disulfide bridge variants to ensure robust manufacturing of a safe and efficacious product.

## Methods

### Triple digest LC-MS peptide map

This multi-enzyme non reducing peptide map allows characterization of correct and incorrect disulfide bridge structures present in etanercept using mass spectrometry. Samples containing 100 µg of etanercept were transferred into a sodium acetate buffer pH 5.0 and treated with 60 mU of sialidase (Roche) and 4 U pNGaseF (Roche) for 16 hours at 37 °C to remove sialic acid and N-glycans, respectively. Samples were subsequently transferred into 50 mM Tris buffer pH 8.0 containing 0.5% RapiGest SF (Waters) and 50 mM iodoacetamide and denatured for 30 min at 50 °C. Samples were then buffer exchanged into 50 mM Tris buffer pH 8.0 containing 0.1% Rapigest SF solution and digested with 0.1 µg/µL AspN (Roche), 1 µg/µL Trypsin (Promega Gold) and 1 µg/µL chymotrypsin (Roche) for 16 hours at 37 °C. Digestion was terminated by addition of formic acid and acetonitrile to a final concentration of 0.3% and 3%, respectively. To achieve optimal peak resolution, an Ascentis Express Peptide ES-C18 column was equilibrated for 15 min with 0.0375% ammonium formate in HPLC water. Following sample application, peptides were eluted from the column by applying a gradient of 0.0345% ammonium formate and 90% acetonitrile in water over a 110 min time frame. A Q Exactive mass spectrometer (Thermo scientific) was used for identification of the disulfide bridge peptides in the sample (see Supplementary Table S1 for details).

### Non-reducing peptide map

This non reducing peptide map allows quantitation of a tryptic peptide T7 containing the incorrectly disulfide bridge C_78_-C_88_ relative to an internal peptide standard T27 in etanercept samples. Samples containing 150 µg of etanercept were denatured using 0.1% RapiGest SF (Waters) in Tris buffer pH 8.0 containing 50 mM iodoacetamide at 50 °C for 40 minutes. Samples were subsequently buffer exchanged into 50 mM MES buffer containing 0.05% RapiGest SF pH 6.0 and digested with 12 µg Trypsin (Promega Gold) for 17 hours at 37 °C. Digestion was terminated by addition of formic acid and acetonitrile to a final concentration of 0.3% and 3%, respectively. Separation was performed using an Ascentis Express Peptide ES-C18 column. To achieve optimal peak resolution, the column was flushed for 15 min with buffer B (0.1% TFA in 90% Acetonitrile and 10% HPLC water) and 10 min with buffer A (0.1% TFA in HPLC water) before use. Peptides were eluted using a gradient of 0–16% buffer B over 25 min, 18% buffer B at 28 min and 100% buffer B at 33 min. The UV absorption at 215 nm was recorded by a UV detector. Example chromatograms and mass spectra are shown in Supplementary Figs S2–S4. Examples of tests used demonstrate that the method is scientifically sound and fit for purpose are shown in Supplementary Tables S2 and S3.

### Analytical hydrophobic interaction chromatography

GP2015 variants were separated according to their hydrophobicity using hydrophobic interaction chromatography (HIC). Samples were diluted by addition of water to a target concentration of 2.0 mg/mL and chromatographically resolved using a Tosoh, TSKgel Buty-NPR column with a gradient consisting of 1.2 M (NH_4_)_2_SO_4_ in 0.1 M phosphate buffer, pH 7.0 and 0.1 M phosphate buffer, pH 7.0. A fluorescence detector with an excitation wavelength of 278 nm and an emission wavelength of 350 nm was used for detection and quantification.

### Potency

Binding and functional neutralization of TNF-α by GP2015 and Enbrel^®^ samples was measured using HEK293 cells stably transfected with an NFκB-luciferase reporter gene. In a microtiter plate, 20,000 cells per well were stimulated with 4 ng/mL TNF-α in the presence of graded amounts of GP2015 or Enbrel^®^. After overnight incubation, cells were lysed and luciferase activity was quantified in the lysates using a luminogenic substrate. The potency of GP2015 or Enbrel® was determined by comparison to a reference standard and relative potency was calculated using a parallel line assay according to the European Pharmacopoeia.

### Serum-like reducing conditions

Incorrect disulfide bridges of Enbrel® and GP2015 drug product, drug substance, process intermediates and late eluting HIC fractions were reshuffled by incubation under physiologically relevant cysteine levels. Samples were diluted to 4 mg/ml in redox-buffer (0.83 mM cysteine, 0.17 mM cystamine in 200 mM Tris buffer pH 8.0) and incubated for 48 hours at 2–8 °C. Samples were subsequently dialyzed in citrate buffer (25 mM citrate pH 6.3) using Slide-A-Lyzer 10000 MW dialysis cassettes (Thermo Scientific). Dialysis buffer was exchanged three times every 20 minutes. Dialyzed samples were diluted to 2 mg/ml with citrate buffer prior to analysis.

### X-ray crystallography

The receptor domain of etanercept was released by papain digestion of the full-length dimeric fusion protein. The Fc fragments were removed by a Protein A column and the receptor domain was incubated with TNFα. The yielded complex was further purified on a S-200 gel filtration column and fractions containing the TNFα etanercept receptor complex was additionally concentrated using a 50 kDa Vivascience ultrafiltration device. Obtained crystals were flash-frozen and measured at a temperature of 100 K. The X-ray diffraction data sets were collected for GP2015 and Enbrel® at the Swiss Light Source (SLS, Villigen, Switzerland) using cryogenic conditions. Crystals belong to space group H 3 2. The data were processed using the programs XDS and XSCALE.

## Electronic supplementary material

Supplementary Information
