# Supplementary material for: The structure-function relationship of disulfide bonds in etanercept
Source: Sci Rep. 2017 Jun 21;7:3951. doi: 10.1038/s41598-017-04320-5 (PMC5479810; doi:10.1038/s41598-017-04320-5)
Supplement: Supplementary file 1 — Supplementary Information [file 41598_2017_4320_MOESM1_ESM.pdf]

Title: The structure-function relationship of disulfide bonds in etanercept

Authors

William C. Lamanna[1], Robert Ernst Mayer[2], Alfred Rupprechter[2], Michael Fuchs[2], Fabian Higel[3], Cornelius Fritsch[4], Cornelia Vogelsang[2], Andreas Seidl[3], Hansjoerg Toll[1], Martin Schiestl[1] and Johann Holzmann[2,*]

Affiliation

[1] Sandoz Biopharmaceuticals, Sandoz GmbH, Biochemiestraße 10, 6250, Kundl, Austria

[2] Technical Development Biosimilars, Biologics Technical Development and Manufacturing, Novartis, Sandoz GmbH, Biochemiestraße 10, 6250, Kundl, Austria

[3] Technical Development Biosimilars, Biologics Technical Development and Manufacturing, Novartis, Hexal AG, Keltenring 1+3, 82041, Oberhaching, Germany

[4] Novartis Pharma AG, Klybeckstrasse 141, CH-4057, Basel, Switzerland

Correspondance: johann.holzmann@novartis.com

SUPPLEMENTARY INFORMATION

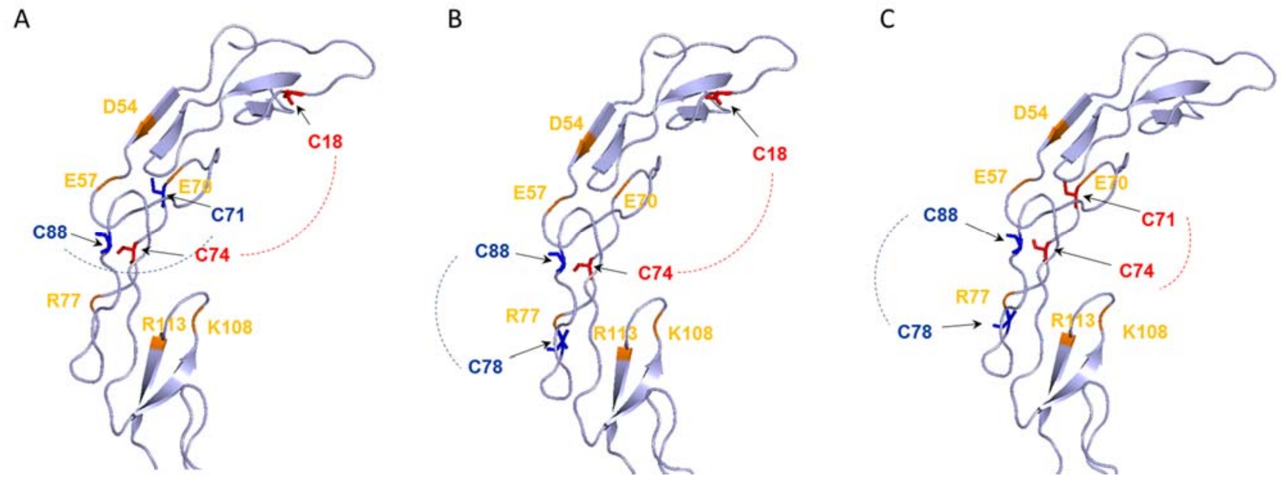

Supplementary Figure 1: Assessment of the structural proximity of cysteins involved in incorrect disulfide bridging within the TNFα binding domain

To enable better assessment of the structural proximity of cysteins found to be involved in incorrect disulfide bridging within the TNFα binding domain, three structural views with different combinations of incorrect disulfide bridges of the TNF-receptor domain are shown (panels A, B and C). Amino acids involved in the binding of TNFα are highlighted in orange. Cysteine pairs involved in the formation of incorrect disulfide bridges are colored in blue and red. The dashed lines indicate distances between the cysteines and provide an impression of the structural changes the TNF-receptor must undergo to allow formation of these incorrect disulfide bridges. Such structural changes would be expected to influence the position of amino acids involved in TNFα binding and in turn the ability to bind TNFα.

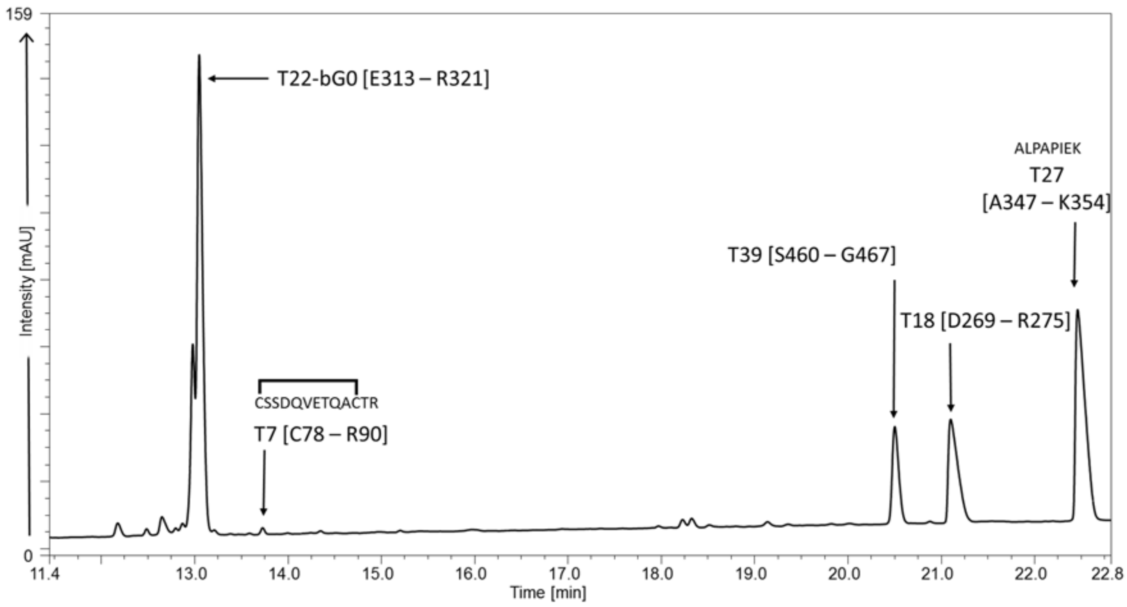

Supplementary Figure 2: Example non reducing peptide map chromatogram

The example non reducing peptide map chromatogram shows the identity of the incorrect C_{78}-C_{88} disulfide bridge peptide T7 and the internal peptide standard T27 in a GP2015 drug substance (DS) sample with low amounts on incorrect disulfide bridging. The identities of T7, T27 and additional major peaks in the chromatogram were determined by mass spectrometry and the respective amino acid sequence for these peaks is given in brackets. For clarity, the peptide sequence of T7 and T27 is additionally represented using the full single letter code and the disulfide bridge in T7 is indicated. Trypsin “T” numbers were assigned arbitrarily. Peptide T22 contains a bG0 N-glycan as indicated.

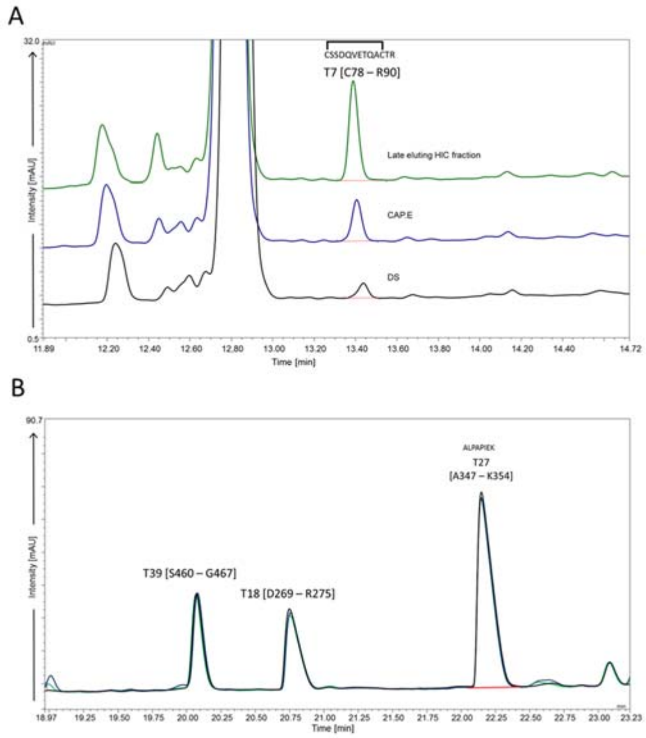

Supplementary Figure 3: Examples of non reducing peptide map detection of incorrect disulfide bridging

The quantitation of incorrect disulfide bridging using the non reducing peptide map is based on the abundance of the T7 peptide containing the incorrect disulfide bridge C_{78}-C_{88} relative to the internal peptide standard T27. Panel A shows the change in abundance of peptide T7 in a representative GP2015 drug substance (DS) sample (black), in a capture eluate (CAP.E) process intermediate sample (blue), and in a late eluting HIC fraction sample (green). Panel B demonstrates that the abundance of the internal peptide standard T27, along with that of additional internal peptides T39 and T18, in these same samples remains unchanged. The identities of major peaks in these chromatograms were verified by mass spectrometry and their respective amino acid sequences are indicated in brackets. For clarity, the peptide sequence of T7 and T27 is additionally represented using the full single letter code and the disulfide bridge in T7 is indicated. Trypsin "T" numbers were assigned arbitrarily.

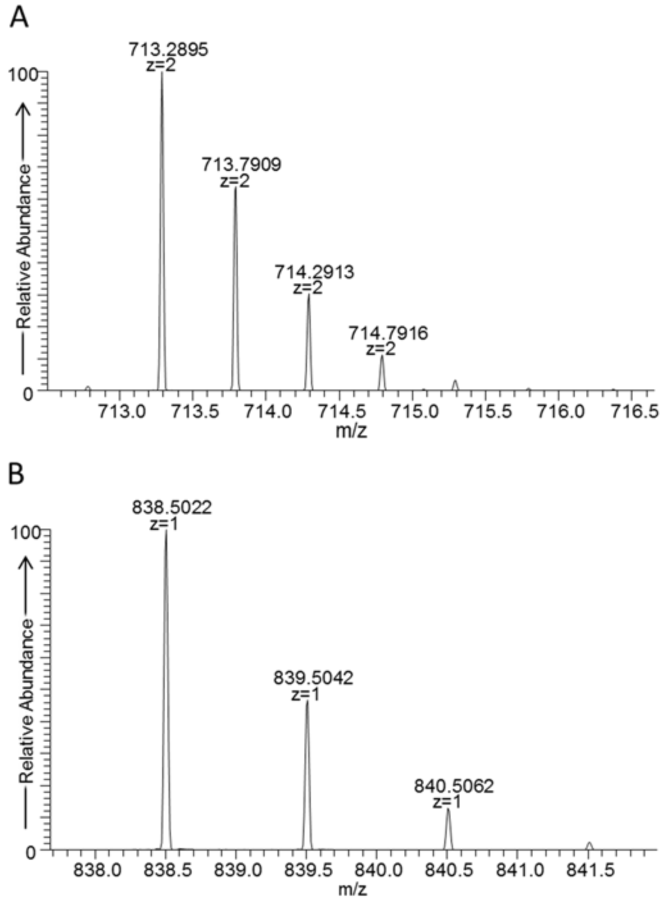

Supplementary Figure 4: Representative mass spectra of relevant non reducing peptide map peptides

The quantitation of incorrect disulfide bridging using the non reducing peptide map is based on the abundance of the T7 peptide containing the incorrect disulfide bridge C_{78}-C_{88} relative to the internal peptide standard T27. The identity of these peptides was verified using a Q Exactive mass spectrometer (Thermo Scientific) and representative mass spectra of T7 and T27 are shown in panels A and B, respectively. The theoretical monoisotopic mass of T7 in the 2^+ charge state is 713.2903 and the theoretical monoisotopic mass of T27 in the single charge state is 838.5033. All peptides were identified within 5 ppm of the theoretical mass

Peptide	Disulfide Bridge	Theoretical mass [charge state]
**Correct disulfide bridge peptides**		
APEPGSTCR-CCSK-CTK	C_{18}-C_{31}, C_{32}-C_{45}	568.2387 [3+]
CSPGQHAK-DTVC	C_{35}-C_{53}	631.2686 [2+]
DSCE-NWVPECLSCGSR-DQVETQACTR	C_{56}-C_{71}, C_{74}-C_{88}	983.402 [3+]
DSCE-NWVPECL	C_{56}-C_{71}	655.755 [2+]
SCGSR-DQVETQACTR	C_{74}-C_{88}	828.8569 [2+]
CSS-ICTCRPGWY-LCAPL	C_{78}-C_{96}, C_{98}-C_{115}	952.9117 [2+]
CALSK-QEGCR	C_{104}-C_{112}	555.7552 [2+]
CRPGF-DVVCKPCAPGTF-DICRPH	C_{121}-C_{139}, C_{142}-C_{157}	850.7218 [3+]
QICNVVAIPG-DAVCT	C_{163}-C_{178}	759.8682 [2+]
**Incorrect disulfide bridge peptides**		
APEPGSTCR-SCGSR	C_{18} –C_{74}	712.3063 [2+]
NWVPECL-DQVETQACTR	C_{71}-C_{88}	1004.4486 [2+]
CSS-DQVETQACTR	C_{78}-C_{88}	722.2956 [2+]
NWVPECLSCGSR	C_{71}-C_{74}	674.7923 [2+]

Supplementary Table 1: Correct and incorrect disulfide bridging identified using the triple digest LC-MS peptide map

The most abundant disulfide bridge peptides identified using the triple digest LC-MS peptides map are indicated using single letter amino acid code and individual peptides connected by a disulfide bond are indicated using hyphens. The theoretical monoisotopic mass of the most abundant charge state for each peptide is indicated. All monoisotopic masses used to identify these peptides were within 5 ppm of the theoretical mass and the amino acid sequences were verified by MS/MS using a Q Exactive mass spectrometer (Thermal Scientific).

GP2015 drug substance samples				Capture eluate process intermediate samples			
**Sample (number)**	**Etanercept amount**	**Relative % T7**	**RSD**	**Sample (number)**	**Etanercept amount**	**Relative % T7**	**RSD**
GP2015 DS (1/6)	100.5 µg	1.3	4.3 %	CAP.E (1/6)	100.5 µg	3.6	1.6 %
GP2015 DS (2/6)	100.5 µg	1.3		CAP.E (2/6)	100.5 µg	3.6	
GP2015 DS (3/6)	100.5 µg	1.4		CAP.E (3/6)	100.5 µg	3.6	
GP2015 DS (4/6)	100.5 µg	1.3		CAP.E (4/6)	100.5 µg	3.6	
GP2015 DS (5/6)	100.5 µg	1.3		CAP.E (5/6)	100.5 µg	3.7	
GP2015 DS (6/6)	100.5 µg	1.4		CAP.E (6/6)	100.5 µg	3.5	
GP2015 DS (1/6)	150 µg	1.3	4.6 %	CAP.E (1/6)	150 µg	3.5	3.0 %
GP2015 DS (2/6)	150 µg	1.2		CAP.E (2/6)	150 µg	3.5	
GP2015 DS (3/6)	150 µg	1.4		CAP.E (3/6)	150 µg	3.7	
GP2015 DS (4/6)	150 µg	1.3		CAP.E (4/6)	150 µg	3.5	
GP2015 DS (5/6)	150 µg	1.3		CAP.E (5/6)	150 µg	3.7	
GP2015 DS (6/6)	150 µg	1.2		CAP.E (6/6)	150 µg	3.7	
GP2015 DS (1/6)	199.5 µg	1.1	9.1 %	CAP.E (1/6)	199.5 µg	3.4	8.6 %
GP2015 DS (2/6)	199.5 µg	1.1		CAP.E (2/6)	199.5 µg	3.5	
GP2015 DS (3/6)	199.5 µg	1.3		CAP.E (3/6)	199.5 µg	3.6	
GP2015 DS (4/6)	199.5 µg	1.3		CAP.E (4/6)	199.5 µg	3.5	
GP2015 DS (5/6)	199.5 µg	1.3		CAP.E (5/6)	199.5 µg	3.3	
GP2015 DS (6/6)	199.5 µg	1.4		CAP.E (6/6)	199.5 µg	4.2	

Supplementary Table 2: Repeatability testing of the non reducing peptide map method

To assess whether the precision and robustness of the non reducing peptide map method is suitable for its intended purpose, the ability of the assay to repeatedly determine the relative amount of incorrectly bridged T7 peptide in GP2015 drugs substance (DS) and in capture eluate (CAP.E) process intermediate sample was tested. The indicated samples were analyzed at 67%, 100% and 133% of the amount of etanercept specified in the method (150 µg). The amount of trypsin used in the digest was not adjusted for the different concentrations of sample being tested. Sample preparation and digestion was individually performed six times per concentration and the amount of incorrect disulfide bridge C_{78}-C_{88} was quantified as per method by calculating the percent of T7 peptide relative to the internal standard T27. The relative standard deviation (RSD) for each sample set is given.

Assessment of average % T7			Assessment of recovery			
**Sample (number)**	**Relative % T7**	**Average % T7**	**Mixture (ratio)**	**Relative % T7**	**Recovery**	**RSD of Recovery**
HIC.E (1/3)	1.0	1.0	HIC.E : late eluting HIC (5:1)	2.3	107 %	2.0 %
HIC.E (2/3)	1.0		HIC.E : late eluting HIC (5:1)	2.2	104 %	
HIC.E (3/3)	1.0		HIC.E : late eluting HIC (5:1)	2.3	108 %	
Late eluting HIC (1/3)	7.7	7.8	HIC.E : late eluting HIC (1:1)	4.8	109 %	4.1 %
Late eluting HIC (2/3)	7.8		HIC.E : late eluting HIC (1:1)	4.9	111 %	
Late eluting HIC (3/3)	8.0		HIC.E : late eluting HIC (1:1)	4.5	103 %	
			HIC.E : late eluting HIC (1:5)	6.5	98 %	5.3 %
			HIC.E : late eluting HIC (1:5)	7.1	107 %	
			HIC.E : late eluting HIC (1:5)	6.5	97 %	

Supplementary Table 3: Accuracy testing of the non reducing peptide map method

To assess whether the accuracy of the non reducing peptide map method is suitable for its intended purpose, the ability to detect and recover the expected amount of incorrectly bridged T7 peptide after mixing samples with known amount of incorrect disulfide bridging was assessed. To this end, HIC eluate (HIC.E) sample was mixed with late eluting HIC sample containing high amounts of incorrect bridged T7 peptide at ratios of 5:1, 1:1 and 1:5 prior to digestion. Samples were prepared in triplicate, digested according to the method and the percent of T7 peptide relative to the internal standard T27 was assessed. To calculate the theoretical amount of T7 peptide expected in the mixed samples, the relative amount of T7 in the HIC.E and late eluting HIC samples was assessed in triplicate and the average percent T7 was used for calculation. The percent recovery reflects the actual amount of T7 detected in the mixed samples versus the theoretical amount expected. The relative standard deviation (RSD) of percent recovery is additionally indicated.
